# Intelligent personalized shopping recommendation using clustering and supervised machine learning algorithms

**DOI:** 10.1371/journal.pone.0278364

**Published:** 2022-12-01

**Authors:** Nail Chabane, Achraf Bouaoune, Reda Tighilt, Moloud Abdar, Alix Boc, Etienne Lord, Nadia Tahiri, Bogdan Mazoure, U. Rajendra Acharya, Vladimir Makarenkov

**Affiliations:** 1 Department of Computer Science, Université du Québec à Montréal, Montreal, QC, Canada; 2 Institute for Intelligent Systems Research and Innovation, Deakin University, Geelong, Victoria, Australia; 3 University of Sherbrooke, Sherbrooke, QC, Canada; 4 School of Computer Science, McGill University, Montreal, QC, Canada; 5 Quebec AI Institute (MILA), Montreal, QC, Canada; 6 Department of Electronics and Computer Engineering, Ngee Ann Polytechnic, Singapore, Singapore; 7 Department of Biomedical Engineering, School of Science and Technology, SUSS University, Singapore, Singapore; 8 Department of Biomedical Informatics and Medical Engineering, Asia University, Taichung, Taiwan; Hanyang University, KOREA, REPUBLIC OF

## Abstract

Next basket recommendation is a critical task in market basket data analysis. It is particularly important in grocery shopping, where grocery lists are an essential part of shopping habits of many customers. In this work, we first present a new grocery Recommender System available on the MyGroceryTour platform. Our online system uses different traditional machine learning (ML) and deep learning (DL) algorithms, and provides recommendations to users in a real-time manner. It aims to help Canadian customers create their personalized intelligent weekly grocery lists based on their individual purchase histories, weekly specials offered in local stores, and product cost and availability information. We perform clustering analysis to partition given customer profiles into four non-overlapping clusters according to their grocery shopping habits. Then, we conduct computational experiments to compare several traditional ML algorithms and our new DL algorithm based on the use of a gated recurrent unit (GRU)-based recurrent neural network (RNN) architecture. Our DL algorithm can be viewed as an extension of DREAM (Dynamic REcurrent bAsket Model) adapted to multi-class (i.e. multi-store) classification, since a given user can purchase recommended products in different grocery stores in which these products are available. Among traditional ML algorithms, the highest average F-score of 0.516 for the considered data set of 831 customers was obtained using Random Forest, whereas our proposed DL algorithm yielded the average F-score of 0.559 for this data set. The main advantage of the presented Recommender System is that our intelligent recommendation is personalized, since a separate traditional ML or DL model is built for each customer considered. Such a personalized approach allows us to outperform the prediction results provided by general state-of-the-art DL models.

## 1 Introduction

Grocery shopping is a common activity that involves several important factors such as time, budget, and purchasing pressure [1]. In this context, well-conceived grocery lists can be an efficient planning and budgeting tool. Several studies have indicated that a majority of modern customers rely on a written, mental, or digital grocery list [2, 3] in order to assist them in their shopping. Furthermore, the same studies have also revealed that consumers generally had growing interest in applications that helped them interactively manage their grocery lists, while informing them about products prices and special offers.

Typically, grocery retailers propose new specials every week to attract new customers and improve sales and profits. For example, Walters and Jamil [4] have shown that in a regular grocery shopping trip involving cross-category products, 39% of the items in a customer’s basket were special offers. These authors also concluded that about 30% of surveyed customers were highly influenced by different coupons and specials.

While special prices sometimes allow customers to make significant savings, thousands of them are usually released every week, often leading to huge information overload. This makes the task of selecting the most advantageous offers for a given customer an extremely challenging one [5].

With the development of online shopping, recent advancements in machine learning techniques, and favorable reactions of many customers to user-friendly applications aiming at improving their shopping experience, the development of an online recommender grocery shopping system able to provide valuable individual recommendations seems to be a very relevant task. MyGroceryTour (http://mygrocerytour.ca) is a good example of such a recommender system. MyGroceryTour is a Canadian shopping database and website that allows users to manage their grocery lists based on available weekly promotions in most major grocery stores located in their area [6].

**Fig 1:**
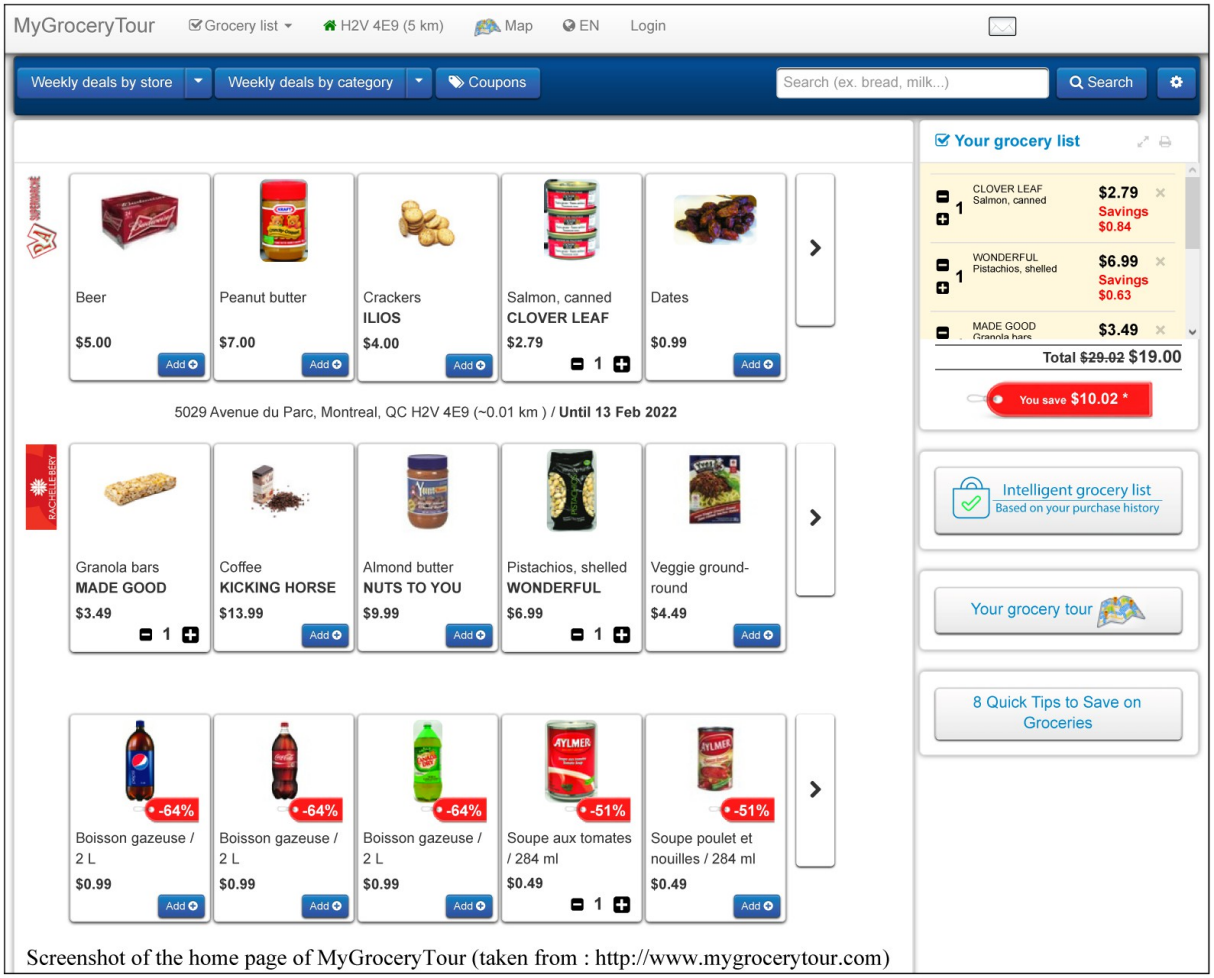
Home page of the MyGroceryTour website. Specials from different stores located near a specified postal code or address (in Canada) can be added to the user’s basket.

One of the main purposes of our study is to present a new ML-based recommender system for grocery shopping based on the MyGroceryTour users’ purchase histories, profiles, preferences and available weekly specials in order to assist them in creating cost-effective personalized weekly grocery lists (see Fig 1).

Our main contributions are the following:

1. We present our novel personalized Recommender System available on the MyGroceryTour platform;
2. We perform a clustering analysis to partition Canadian customers into non-overlapping clusters according to their grocery shopping habits;
3. We define the quantity-based fidelity ratio and the price-based fidelity ratio features to characterize the customer’s shopping behaviour;
4. We describe and apply a new deep learning RNN-GRU model (i.e. an extended multi-class DREAM model) to predict whether a given product should be included in the user’s multi-store basket, and to recommend the store where the purchase should be made (if any);
5. Our clustering and supervised machine learning analyses suggest that different prediction models (i.e. traditional ML models or DL models) should be used for different groups of customers.

The paper is organized as follows: We first summarize the related work in the field. Then, we present the main features of the MyGroceryTour platform and the associated customer-based recommender system. This is followed by the data and methodology descriptions, including the application of clustering, traditional machine learning, and deep learning algorithms. Our main results are then described and discussed in the Results and Discussion section, which is followed by our main conclusions.

## 2 Related work

Recommender systems (RS) [7] have been an increasingly important field of study since the first research papers on Collaborative Filtering in the mid-90s [8–10], and the expansion of e-commerce and online shopping [11]. Recommender systems include algorithms and software aiming at providing users with personalized items recommendations to help them overcome the data overload issue and to assist them in decision-making processes. The recommended items represent the output of the recommender system while their nature may vary depending on the context; among others, the items can include movies, songs, retail products, or online documents [7, 12].

Nowadays, several strategies to build recommender systems have been described in the literature. Here, we present the most popular of them, and those related to our case of study.

In their work, Melville and Sindhwani [13] classified RS techniques into three major categories:

- Collaborative filtering (CF) approaches;
- Content-based filtering (CB) approaches;
- Hybrid approaches.

Collaborative filtering is one of the most popular and efficient RS techniques [14, 15]. It is based on the *word-of-mouth* concept and admits that a user trusts another user with similar reasoning and taste. It also makes the assumptions that two similar users have similar interests, and that two similar items have similar ratings [16]. The most common limitations faced by CB methods are the *cold start* and *sparse matrix* issues [17]. The cold start issue is characterized by the lack of initial information regarding a newly introduced user or item, whereas the sparse matrix issue typically occurs when a given user tends to interact with a few items only out of the massive amount of available products [12, 18, 19].

Content-based filtering, on the other hand, tends to recommend items whose features and characteristics are similar to other items in which a given user showed positive interest in the past [20]. This approach requires the use of metadata relative to each considered item what can sometimes represent a challenge.

In an attempt to overcome the limitations of the collaborative filtering and content-based filtering techniques, hybrid approaches, trying to combine both of them, have been introduced. The works of Adomavicius *et al*. [21] as well as, more recently, Lu *et al*. [12] reviewed different methods used in the field of RS, highlighting their pros and cons and giving insight into the future developments in the field.

Recently, several important extensions of traditional RS approaches have been introduced [22–24]. The main of them are as follows:

- Knowledge-based recommender systems (KBRS);
- Context-aware recommender systems (CARS);
- Demographic-based recommender systems (DBRS).

Knowledge-based recommender systems [25, 26] can be efficiently used for recommending highly customized products (i.e. real estate or automobiles). Unlike classical methods such as CF or CB, KBRS looks to obtain explicit user requirements by the direct solicitation, allowing the user to have more control over the recommendation while building interactive feedback.

Context-aware recommender systems [24, 27] rely on multiple sources of information to identify a certain context and to generate more accurate recommendations (e.g., recommending swimsuits instead of winter coats in summer).

Finally, demographic-based recommender systems [28] group users based on their available demographic attributes (i.e., age, gender, location), assuming that people within the same group (neighborhood) rate items similarly. This approach has originally been introduced to improve the quality of recommendations but, it has also proved to be useful for solving the cold start problem [29].

Let us now recall some recent works addressing the issue of next grocery basket recommendation. Yu *et al*. [30] introduced an efficient model, called Dynamic REcurrent bAsket Model (DREAM), based on recurrent neural networks. One of the main advantages of DREAM is that it is not only able to learn a dynamic representation of a user but also takes into account global sequential features among baskets. However, the original DREAM model of Yu *et al*. was designed to perform binary classification only. For each available product, the model generates a probability score accounting for the probability that this product will be included in the next basket purchased by a given customer. Nevertheless, DREAM cannot provide predictions in a multi-store (i.e. multi-class) context, consisting in predicting the store where the recommended product should be bought. Moreover, in their work, Yu *et al*. did not consider some important features such as product prices, product availability, and weekly specials offered in local stores. This motivated us to generalize the original DREAM model to a multi-class classification task to predict both whether a given product should be included in the customer’s next basket and in which store the purchase should be made (for more details, see the Materials and methods section).

Che *et al*. [31] described a new prediction method using attention-based recurrent neural networks to detect and model both inter- and intra-basket relationships. The authors proposed to consider all available user’s baskets to model his/her long-term preferences, whereas the intra-basket attention model was intended to act on the item level in his/her most recent baskets to predict the user’s behavior and current short-term preferences. Through their adaptive attention mechanism, Che *et al*. were able to outperform state-of-the-art methods for next basket recommendation, although their method applies only in a binary classification context.

Faggioli *et al*. [32] used the recency factor to predict the consumer’s next grocery basket applying a CF-based prediction method under a general top-n recommendation framework. To show the efficacy of their method, the authors compared it with some state-of-the-art CF models.

Content-based recommendations were also shown to be effective in the field of next basket and grocery coupon recommendation. In this context, Xia *et al*. [33] proposed a tree-based CB model for coupon recommendations. These authors streamlined the coupon selection process in order to personalize the recommendation and increase the clickthrough rate. Using the random forest and XGBoost classifiers, Xia *et al*. were able to improve the estimated coupon click rate from 1.20% to 7.80%.

Moreover, Prokhorenkova *et al*. [34] described and tested a new statistical method based on the Yandex CatBoost model to predict whether a given customer is sensible to purchase some selected products. Dou [35] considered real unbalanced shopping data from an e-commerce platform and used the CatBoost model to predict whether customers will buy or not some available products. The method proposed by Dou was able provide the prediction accuracy of 88.51%.

Lee *et al*. [36] proposed to use recurrent neural networks instead of collaborative filtering techniques to create a multi-period product recommender system related to an online food market. The system introduced by Lee *et al*. is able to recommend products by multiple periods in a time sequence. The authors showed that the proposed recommender system provided a higher performance in accuracy and diversity in a multi-period perspective than CF-based systems. Moreover, the proposed system also showed a robust behavior in terms of consumers’ purchasing orders and repetitive purchase patterns.

Zheng and Ding [37] proposed a personalized recommendation system based on an Immersive Graph Neural Network (IGNN), which is intended to increase the marketing quantity of various commodities, to improve users’ shopping experience, promote sales, and thus motivate the market development. The authors considered an immersive marketing environment using deep learning and graph neural network models. However, as suggested by the authors, the proposed recommendation system was not verified in practical applications. Thus, the impact of the presented model on real users was not assessed.

Finally, Tahiri *et al*. [6] have recently proposed to use both recurrent and feedforward neural networks that were combined to non-negative matrix factorization and gradient boosting trees in order to build intelligent grocery baskets for the users of the MyGroceryTour platform. Tahiri *et al*. considered different features and much less real customers (compared to our study) to describe the behavior of the MyGroceryTour users. Their best F-score result of 0.37 was obtained when their general prediction model was applied to an augmented dataset. However, in their work, Tahiri *et al*. did not perform any clustering analysis and did not consider different categories of customers. As we will see in the next sections, this kind of analysis is very important for improving the prediction performance. Moreover, Tahiri *et al*. did not compare the results generated by their DL model with those provided by traditional ML algorithms. Such a comparison is crucial when the data set at hand is rather small. Finally, the DL model introduced by these authors is not personalized as the same model architecture was used for all customers considered.

In their paper, Gupta and Shrinath [38] presented a Collaborative Filtering-based model tailored to overcome the cold start problem. To achieve that, the authors propose to compute the weighted sum of four different features. The first of them is the items rating obtained using Weighted Non-negative Matrix Factorization, followed by Affinity Propagation technique. The three other ratings are graph-related similarity measures based on the users metadata as well as on their purchasing habits. Gupta and Shrinath reported that their model outperformed the existing approaches based on Hit Ratio and Normalized Discounted Cumulative Gain.

Li *et al*. [39] suggested several novel metrics to measure the repetition/exploration ratio and performance of next basket recommender systems. They compared and analyzed the results of state-of-the-art next basket recommendation models on three real-world datasets. Their study was conducted with a focus on their new metrics in order to help illustrate the scope of the current state of research and explain the progress provided by the existing approaches as well as the reasons behind the achievements claimed by the studied methods. Li *et al*. indicated that future research on next basket recommendation should consider an analysis of repetition and exploration behavior to gain useful insights and help to design unbiased models.

Le *et al*. [40] proposed a framework to model user’s basket sequences. Their hierarchical network model, called Beacon and based on an LSTM architecture, consists of three main components, taking as input a basket sequence and a correlation matrix. The basket encoder component produces correlation-sensitive basket representations after capturing intra-basket item correlations. The sequence of basket representations is then used as input for a sequence encoder to extract inter-basket sequential associations. The output from this component is associated with the correlation matrix, and both are used by the predictor component to produce the correlation-sensitive next basket. Therefore, Le *et al*. took into account the correlative dependencies between items to enhance the representation of individual baskets as well as the overall basket sequence.

## 3 Materials and methods

### 3.1 Mygrocerytour website

MyGroceryTour is a Canadian grocery information platform available in English and French. The main purpose of MyGroceryTour is to provide users with up-to-date information on the best grocery deals offered by major grocery retailers in their area, allowing them to compare the available products and to create personalized weekly grocery lists based on the provided insights.

**Fig 2:**
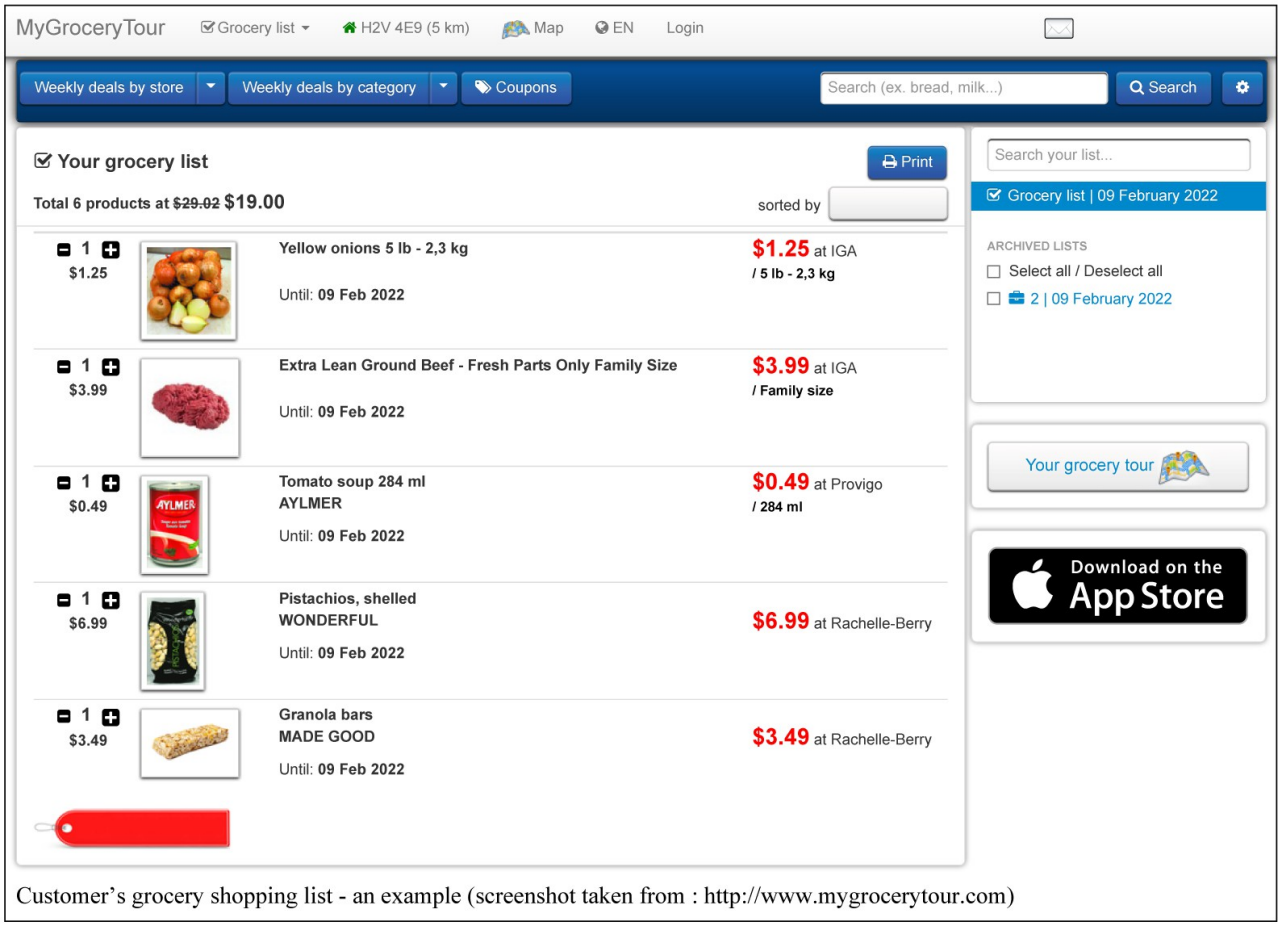
Example of a customer’s grocery shopping list on the MyGroceryTour website.

**Fig 3:**
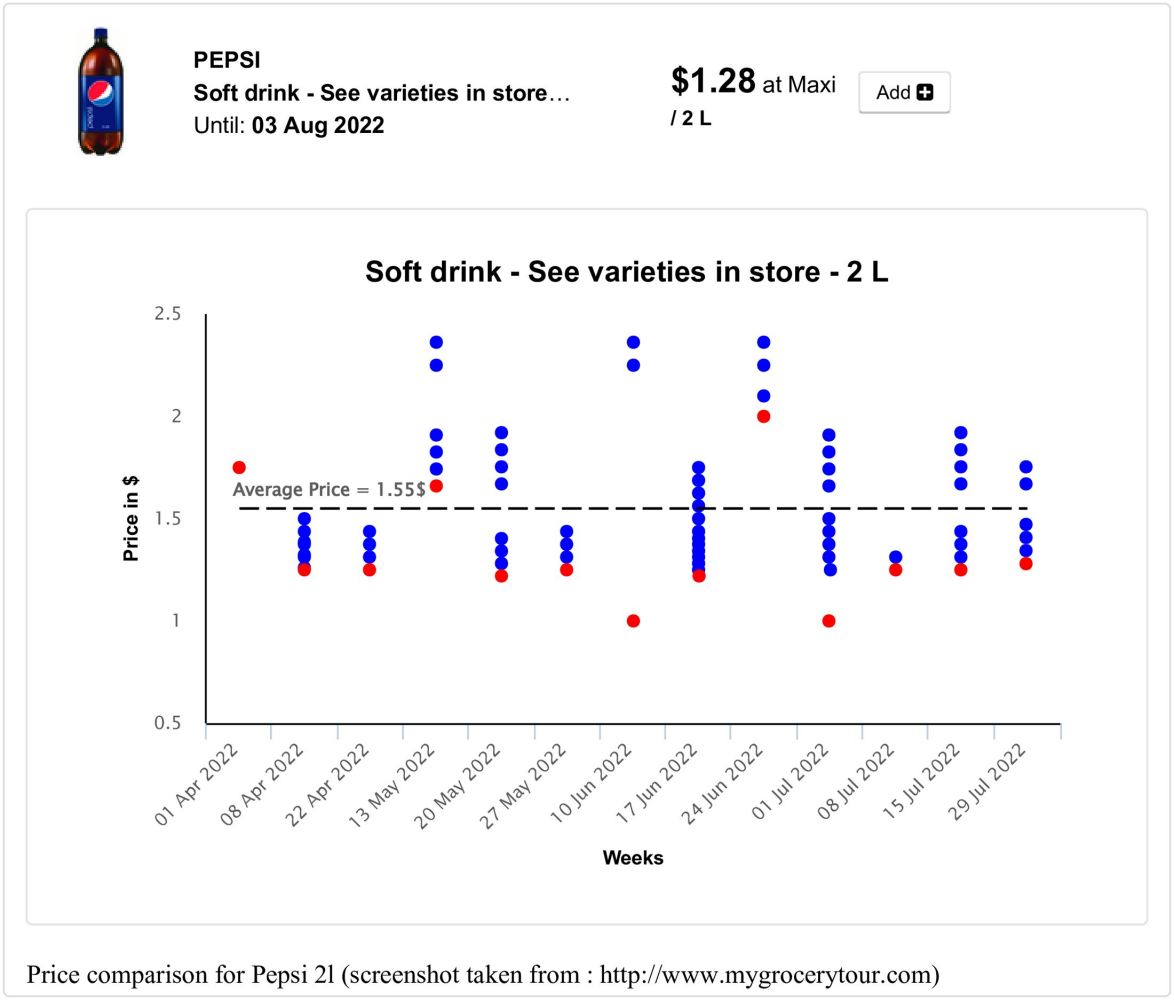
Price comparison for a selected product (Pepsi 2l) in local stores of Montreal over a 3-month period displayed on the MyGroceryTour website. Red dots represent the best weekly deals for the product selected. Blue dots represent other prices of the product available during a given week. Additional price and store information becomes available when touching a dot.

**Fig 4:**
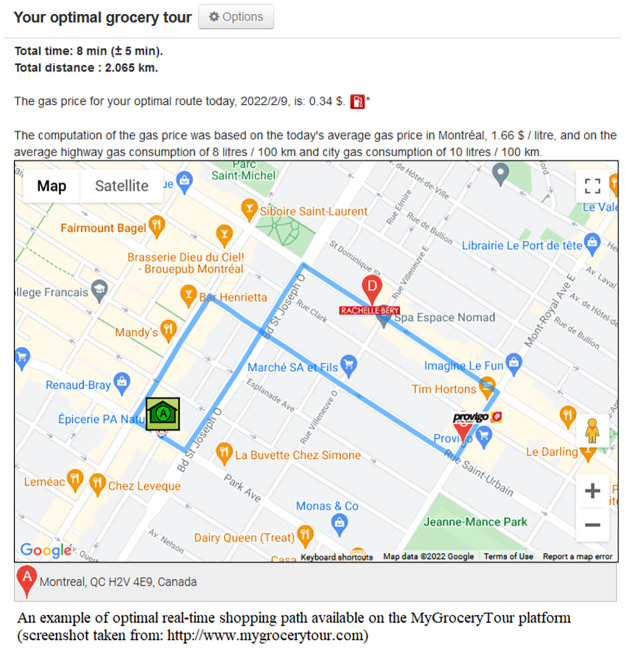
An optimal real-time shopping path, based on the user’s weekly shopping list, displayed on the MyGroceryTour platform.

**Fig 5:**
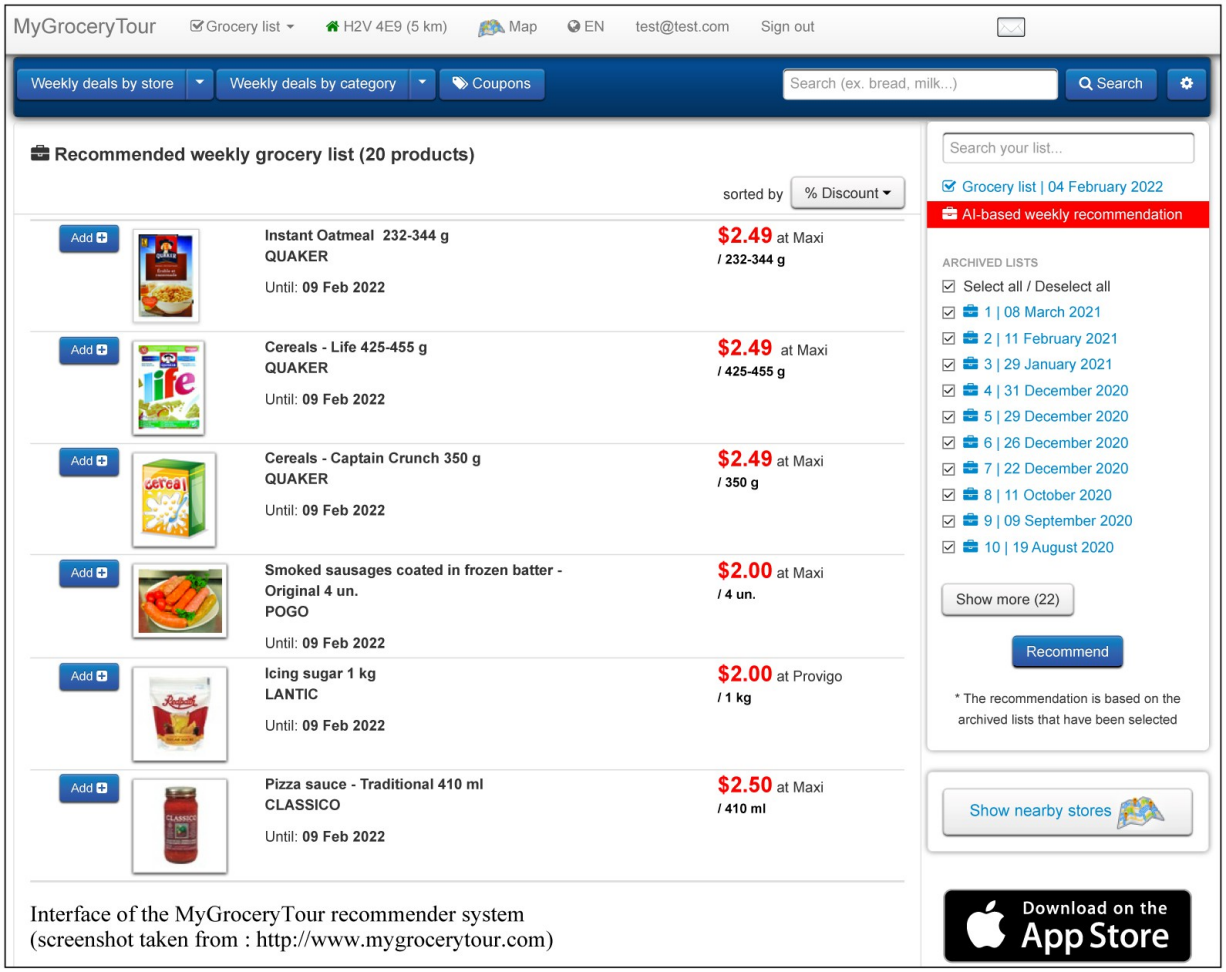
Interface of the MyGroceryTour recommender system.

The main features of the MyGroceryTour platform are as follows. It allows users to:

1. Search and compare grocery deals in the user’s favorite local grocery stores;
2. Create, save, manage and print weekly grocery shopping lists (see Fig 2);
3. Display a map of local grocery stores and pharmacies available for a given postal code or address;
4. Compare the price of a selected product in local stores over a 3-month period (see Fig 3);
5. Find popular Canadian coupons;
6. Display the optimal shopping path based on the user’s shopping list (see Fig 4);
7. Receive email alerts when the user’s favorite products go on sale;
8. Create personalized intelligent grocery lists following a recommendation by machine learning algorithms (see Fig 5). This recommendation is based on the user’s purchase history, the availability of the user’s favorite products and the weekly specials offered in local grocery stores and pharmacies.

A MyGroceryTour feature allow customers to compare prices of a selected product at different stores, making it much easier to identify real specials and opportunities. Customers have the possibility to change their search area depending on their geographical position and their needs while displaying the available grocery products (the 1 to 20 kilometer distance, from the user’s home, can be specified). Moreover, the users of MyGroceryTour can easily create, manage and save their grocery lists, and then access them at any time.

While users cannot purchase items from retailers directly through the website, they can add products from different stores to their baskets. Once a weekly grocery list is organized, the system will recommend to the user the optimal shortest path starting at the user’s home, passing by all selected grocery retailers or pharmacies, and ending at the user’s home as well. An efficient algorithm for solving the Generalized Travelling Salesman Problem (GTSP) by Tasgetiren *et al*. [41] has been implemented by our team, taking into account the real-time local traffic information provided by Google Maps API and the geographical position of the closest stores belonging to selected retailers (several stores for a selected retailer can be available in a given area).

The uniqueness of MyGroceryTour is due to the use of the intelligent recommender system allowing the registered users to get personalized weekly grocery recommendations based on the use of the Random Forest and extended RNN-GRU-based DREAM algorithms which yielded the best prediction results in our experiments (see the Results and discussion section). A test account with the following coordinates (login: test@test.com; password: 123456) has been set up. It can be used to test our ML-based recommender system integrated into the MyGroceryTour platform.

### 3.2 Data description

In this section, we present the dataset which was used in our study. We considered 831 users of the MyGroceryTour web platform with varying amounts of saved weekly grocery lists (varying between 3 and 99). All real data considered here were anonymized. The data are available at: https://drive.google.com/file/d/1q-LkWMx5ar-OGlPPLFwSDi-IbLe7ZaIo/view?usp=sharing. The data collection and analysis method complied with the terms and conditions for the source of the data. Grocery lists used in our experiments included grocery products the users planned to buy during a given week (the time period from January 2017 to June 2021 was covered). The following features (i.e, explanatory variables) from the original dataset have been considered in our experiments:

- *user_id* (numerical): unique user identifier;
- *list_id* (numerical): unique shopping list identifier;
- *product_id* (numerical): unique product identifier;
- *category* (categorical): category of the product;
- *price* (numerical): the price of the product;
- *special* (numerical): discount on the product (in %) compared to its regular price;
- *distance_avg* (numerical): average distance between the user’s home and all stores where the product was available;
- *availability* (binary): availability of the product at different stores.

We completed this list of features by an additional *total_bought* feature that represents the total number of times a given product has been bought by all users.

### 3.3 Data normalization

Data normalization is a common practice and an important step in both unsupervised and supervised machine learning [42], and data mining [43]. Data normalization has been proved theoretically and empirically to be an essential step to obtain better predictions from a model [44–46]. Normalization allows one to bring all features to the same scale, making them mutually comparable, thus ensuring stabler learning process and providing better results for both clustering and supervised learning methods, and specifically for gradient-based algorithms. Prior to feeding the data to our models, we also applied a standardization method to our continuous feature (i.e., product’s category), converting it into a numerical vector. We used the *feature_hasher* class from scikit-learn [47, 48] to encode the *category* feature. This class takes strings as input and converts them into numerical vectors using a hash function.

In our study, we used two popular data normalization techniques: z-score and MinMax rescaling [49].

Z-score normalization is a rescaling of data so that the normalized data have a mean of 0 and a standard deviation of 1 (Eq 1):

$$\begin{array}{c} z\left(x_{f}\right)=\frac{x_{f}-\mu_{f}}{\sigma_{f}}, \end{array}$$ (1)

where *z*(*x*_*f*_) is the normalized value, and *x*_*f*_ is the observed original value of feature *f* at a given observation, *μ*_*f*_ is the mean of *f*, and *σ*_*f*_ is the standard deviation of *f*.

The MinMax normalization is carried out using the following formula (Eq 2):

$$\begin{array}{c} x_{f}^{\prime }=\frac{x_{f}-min\left(x_{f}\right)}{max\left(x_{f}\right)-min\left(x_{f}\right)}, \end{array}$$ (2)

where $x_{f}^{\prime }$ is the normalized value and *x*_*f*_ is the observed original value of feature *f* at a given observation, *min*(*x*_*f*_) is the minimum value of feature *f* over all observations, and *max*(*x*_*f*_) is the maximum value of feature *f* over all observations.

### 3.4 Clustering methods

Clustering is part of data analysis aiming at finding homogeneous groups of objects in data. Clustering algorithms are divided according to input data formats and output cluster structure formats. A generic data format is the so-called object-to-feature matrix *X* = (*x*_*if*_), in which the rows *x*_*i*_ (*i* = 1, 2, …, *N*) correspond to given objects (customers in our case) and columns *f* (*f* = 1, …, *F*) correspond to features characterizing those objects (e.g., product’s price, product’s rebate (if on special), product’s category in our case). A generic cluster structure format is a partition of the set of objects in non-overlapping clusters *S*_1_, *S*_2_, …, *S*_*K*_. The number of clusters *K* must be 2 or more, but not too many, so that usually *K* ≪ *N* and clusters are aggregate representations of the data matrix *X*.

Two data clustering methods, K-means [50] and Ward’s [51] algorithms, have been applied in our study.

The cluster structure in *K-means* [50, 52] is specified by a partition *S* of the set of objects into *K* non-overlapping clusters, *S* = {*S*_1_, *S*_2_, …, *S*_*K*_}. Each partition *S* is characterized by the list of objects belonging to each of its clusters *S*_*k*_ (*k* = 1, …, *K*) and the cluster centroids *c*_*k*_ = (*c*_1_, *c*_2_, …, *c*_*K*_). The problem is to find a partition *S* = {*S*_1_, *S*_2_, …, *S*_*K*_} and cluster centroids *c*_*k*_ = (*c*_1_, *c*_2_, …, *c*_*K*_) that minimize the sum of squares criterion. The *K-means algorithm* follows the so-called alternating minimization scheme for finding a *K*-cluster partition that minimizes Criterion (3):

$$\begin{array}{c} W\left(S,c\right)=\sum_{k=1}^{K}\sum_{x_{i}\in S_{k}}\sum_{f=1}^{F}{\left(x_{if}-c_{kf}\right)}^{2}, \end{array}$$ (3)

where *x*_*if*_ is the value of feature *f* at object *x*_*i*_, and *c*_*kf*_ is the value of feature *f* at centroid *c*_*k*_.

Starting with a random initial partition and a set of centroids *c*, it tries to find an optimal partition *S* that minimizes the sum of squares *W*(*S*, *c*) for a given *c*, and then finds the vector *c*′ that minimizes *W*(*S*, *c*). The procedure is repeated till convergence, that is, till *c*′ coincides with *c*. In practice, the method converges fast to a local minimum which depends a lot on the choice of the starting partition.

The *Ward clustering algorithm* [51, 53] follows the so-called agglomerative hierarchical approach. At each step, this algorithm considers a current partition *S* = {*S*_1_, *S*_2_, …, *S*_*K*_} with *K* clusters and their centers *c* = {*c*_1_, *c*_2_, …, *c*_*K*_}, and merges two clusters, *S*_*k*_ and *S*_*l*_, into a new cluster *S*_*kl*_ = *S*_*k*_∪*S*_*l*_, with its center *c*(*k*, *l*) = (*N*_*k*_*c*_*k*_ + *N*_*l*_*c*_*l*_)/(*N*_*k*_ + *N*_*k*_), where *N*_*k*_ and *N*_*l*_ are the cardinalities of clusters *S*_*k*_ and *S*_*l*_, respectively. The clusters to be merged are selected so that the increase in the value of Δ(*k*, *l*) (Eq 4) reaches its minimum over all *k* and *l* (such that *k* ≠ *l*):

$$\begin{array}{c} \Delta (k,l)=W(S(k,l),c(k,l))-W(S,c), \end{array}$$ (4)

where *S*(*k*, *l*) denotes the new partition with *m* − 1 clusters obtained from *S* by merging *S*_*k*_ and *S*_*l*_ (i.e. *S*_*kl*_ = *S*_*k*_∪*S*_*l*_), and *c*(*k*, *l*) denotes the centroid of this new partition. The quantities Δ(*k*, *l*)’s are all positive because the value of Criterion (3) decreases as the number of clusters *K* grows, so that it becomes zero at *K* = *N*. It is not difficult to derive the following formula explicitly expressing Δ(*k*, *l*) through clusters being merged:

$$\begin{array}{c} \Delta \left(k,l\right)=\frac{N_{k}N_{l}}{N_{k}+N_{l}}d\left(c_{k},c_{l}\right), \end{array}$$ (5)

where *d*(*c*_*k*_, *c*_*l*_) is the Euclidean distance between centroids *c*_*k*_ and *c*_*l*_. This formula shows that the square error criterion tends to merge those clusters whose centers are nearest and whose sizes are most unbalanced. The generic Ward clustering algorithm starts with a trivial partition consisting of all singletons being their center, and then merges one by one clusters with the lowest Ward distance (Eq 5) between them till all objects fall into the unique cluster comprising all of them.

### 3.5 Supervised machine learning algorithms

In this section, we present the main characteristics of supervised traditional machine learning and deep learning algorithms used and compared in our work. Their scikit-learn and PyTorch implementations were used in our computation experiments. The obtained results are presented in the Results and Discussion section. Importantly, all machine learning algorithms were applied in a personalized fashion, i.e., a separate machine learning model was constructed for each of the 831 real users considered in our experiments.

*Decision Trees (DT)*: Decision trees are hierarchical models based on a succession of simple decision rules [54]. Each decision tree comprises of a root, nodes, branches and leaves. Each node represents a test of a given attribute, while branches represent the outcome of that test. A decision is taken upon reaching a leaf that corresponds to the predicted class. The decision rules are inferred based on the training data, and the features. A popular approach to building a decision tree is the impurity minimization at each node based on the Gini impurity measure (Eq 6) that aims at reducing the probability of making errors during the classification. The Gini impurity measure is defined as follows:

$$\begin{array}{c} Gini\left(Z\right)=1-\sum_{k=1}^{K}{P_{k}}^{2}, \end{array}$$ (6)

where *Z* is a learning ensemble containing *K* classes, *k* is a given class, and *P*_*k*_ is the proportion of objects belonging to class *k*.

*Random Forest (RF)*: Random Forest is an ensemble learning algorithm processing several decision trees [55]. Each decision tree is built on a sub-sample of the training ensemble with replacement, following a meta-algorithm known as a *bootstrap aggregation* that aims at minimizing the variance and helping avoid the overfitting. The final decision for an observation is taken based on a majority vote between the outcomes of all decision trees. The main advantages of the Random Forest algorithm is that it is known to be resistant to potential outliers as well as to be easily parallelizable.

*Gradient Boosting Tree (GBT)*: Gradient Boosting Trees are an ensemble learning method using decision trees as weak learners and gradient descent optimization (similarly to neural networks) to achieve the best solution for either classification or regression problems [56, 57]. Unlike Random Forest, which relies on bagging, GBT is based, as indicated by its name, on boosting. The algorithm is iterative. It tries to minimize the loss function by sequentially fitting a new tree at each step and correcting the prediction error from the previous steps. There exist different implementations of GBT, and some of them often perform better than others in practice. In this study, we used the scikit-learn, XGBoost and Catboost implementations of the GBT algorithm [58–60]. Previous works in both classical and deep learning literature have shown that ensemble methods (boosting and bagging) of multiple weak learners can drastically improve the performance upon the baseline algorithm. Moreover, boosting tends to outperform bagging on datasets which contain uneven data coverage, hence our choice of XGBoost and CatBoost algorithms.

*Naive Bayes (NB)*: Naive Bayes is the simplest form of a Bayesian network. This probabilistic approach is based on Bayes’ theorem (Eq 7), defined as follows:

$$\begin{array}{c} P\left(y|X\right)=\frac{P(y)P(X|y)}{P(X)}, \end{array}$$ (7)

where *y* is the class and *X* is the set of features. One of the main drawbacks of Naive Bayes is that it makes the strong assumption that all considered features are independent, which rarely occurs in real-life scenarios. Nonetheless, Naive Bayes has been known to provide competitive results in some cases, especially in spam detection and in sentiment analysis [61, 62].

*Support Vector Machines (SVM)*: A Support Vector Machine algorithm attempts to separate a given dataset using a hyperplane. While an infinity of different hyperplanes may exist for that task, SVM chooses the one maximizing the margin between representative observations belonging to each class. These observations are called support vectors [63]. SVM introduces the concept of *soft margin* to deal with outliers or non-linear data, and it permits the algorithm to choose a hyperplane while allowing a few mistakes to obtain a better final separation [64]. However, the data are often not linearly separable even when soft margins are used. In this case, it is possible to transform the data, considering a higher dimensional space which allows for a better class separation. This is achievable through the use of kernel functions such as the radial basis function (rbf) (Eq 8) defined as follows:

$$\begin{array}{c} K\left(x_{i},x_{j}\right)=exp(-\gamma ||x_{i}-x_{j}{\left|\right|}^{2}), \end{array}$$ (8)

where *γ* is the kernel function coefficient set by the user beforehand [65, 66]. The choice of the most appropriate kernel function is usually guided by trial and error.

*Logistic Regression*: Logistic Regression is a simple classification model using a logistic function (Eq 9) to model the probability of all outcomes of a single trial [67]. It is usually of the following form:

$$\begin{array}{c} f\left(x\right)=\frac{1}{1+e^{-(x-\mu )/s}}, \end{array}$$ (9)

where *μ* is a location parameter and *s* is a scale parameter proportional to the variance.

*Multilayer Perceptron (MLP)*: The perceptron is a binary classifier, and the simplest type of neural network [68]. A perceptron’s classification is obtained by calculating the scalar product of the input data (*x*_1_, *x*_2_, …, *x*_*n*_) and the weights (*w*_1_, *w*_2_, …, *w*_*n*_), and by adding a bias *b* to the result. The perceptron then acts as a threshold function providing the final prediction (Eq 10):

$$\begin{array}{c} f\left(x\right)=\{\begin{array}{cc} \;1, & \text{if}\;\;w\cdot x+b\geq 0\text{,}\\ \;0, & \text{otherwise.} \end{array} \end{array}$$ (10)

The training phase of the perceptron consists of finding the optimal values of the weights through an iterative process of comparing the expected output *y* to the predicted output *y*′ until the algorithm converges for the whole dataset.

A Multilayer Perceptron (MLP) is an artificial neural network with an input layer, a hidden layer, and an output layer consisting of interconnected neurons (or perceptrons). Whereas a simple perceptron is only capable of performing binary classification and linear separation of the data, a Multilayer Perceptron is able to capture complex relationships and perform multi-class classification. The data are fed through the input layer, then processed through the hidden layer, while the final output layer gives the final decision. The MLP training phase is similar to that of a simple perceptron—it is also an iterative process aimed at finding the optimal vector of weights **w** by comparing the predicted class *y*′ with the real class *y*. However, considering its more sophisticated nature and the presence of a hidden layer, the MLP relies on backpropagation to handle the errors during the training phase [69].

**Fig 6:**
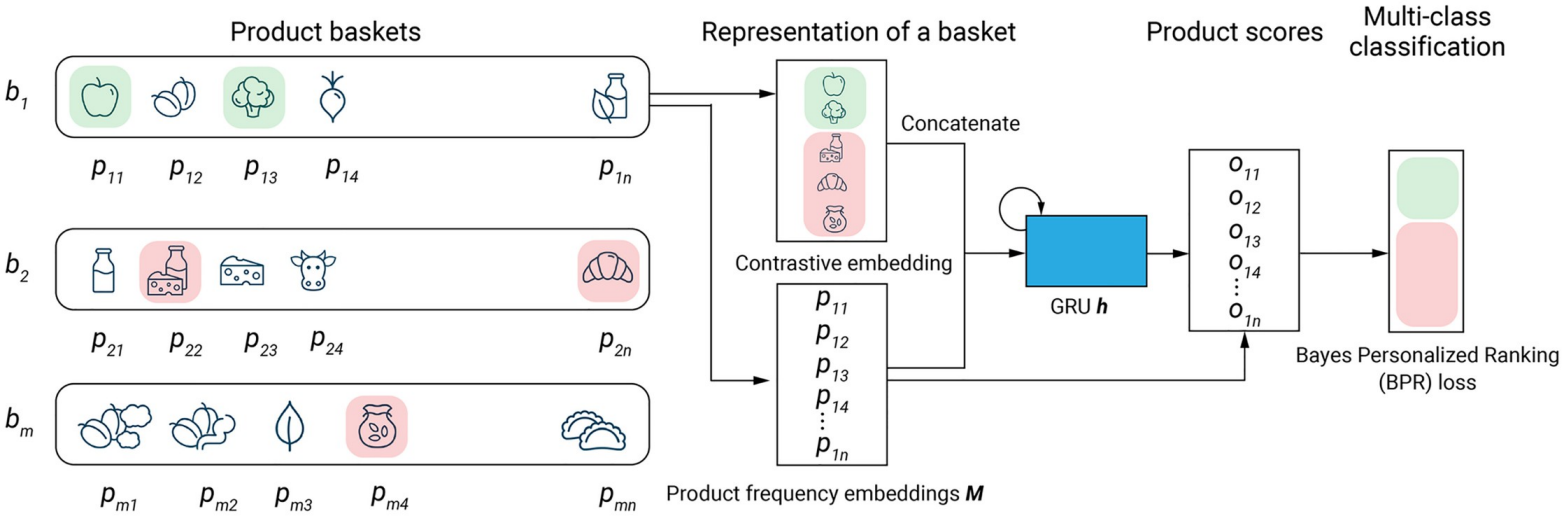
Architecture of our extended DREAM RNN-GRU model for next basket prediction using the Bayes Personalized Ranking (BPR) loss function.

*Proposed RNN-GRU model*: A recurrent neural network (RNN) is a deep learning network designed to embed sequential time-dependent data. In this study, we used a gated recurrent unit (GRU) RNN architecture to represent users’ baskets. Precisely, we generalized the DREAM (Dynamic REcurrent bAsket Model) model proposed by Yu *et al*. [30] to predict the next basket content. Moreover, we used some additional features such as product prices, product availability, and weekly specials offered in local stores, which were not considered by Yu *et al*. We applied some important modifications to the original DREAM model to adapt it to a multi-class classification (only a binary classification problem is considered in [30]). Specifically, we embedded each available product using an *Embedding layer* in PyTorch, which was concatenated with the rest of the features passed through a two-layer perceptron; thus, each product was represented by an augmented vector (see Fig 6 for a schematic view of the proposed model’s architecture).

Precisely, our RNN architecture contained 2 GRU layers of 64 neurons each. The parameter optimization was carried out by the *RMSProp* optimizer in PyTorch. We selected the optimal learning rate using 5-fold cross-validation. To prevent overfitting, we trained the model with a drop-out rate of 0.1.

Since, in this work, we study the benefits of training a single model per user, no explicit user embedding needs to be constructed. Each basket *b*_*i*_ was treated as an arbitrary permutation of products *b*_*i*_ = {*p*_*i*,1_, .., *p*_*i*,*n*_} encoded within the augmented feature space, which was then summarized by the GRU cells into *h*_*i*,*t*_. The hidden embedding *h*_*i*,*t*_ acts as an implicit user representation, since it stores information regarding the user’s shopping baskets.

To obtain the product’s affinity score, i.e. the score proportional to the probability of the product *p*_*i*,*t*_ to be included in a basket containing the products *p*_*i*,1_, ..*p*_*i*,*t*−1_, *p*_*i*,*t*+1_, .., *p*_*i*,*n*_, we multiplied the product embedding matrix *M* by the hidden embedding *h*_*i*,*t*_ (Eq 11):

$$\begin{array}{c} o_{i,t}=M^{T}h_{i,t}. \end{array}$$ (11)

A higher score *o*_*i*,*t*_ indicates that the user is more likely to purchase the corresponding item. The Bayesian Personalized Ranking (BPR) loss was used to approximate and maximize the following probability (Eq 12):

$$\begin{array}{c} p\left(b_{i},v≻v^{\prime }\right)=\sigma \left(o_{i,v}-o_{i,v^{\prime }}\right), \end{array}$$ (12)

where *v* denotes a positive item included in the basket *b*_*i*_, *v*′ denotes a negative item not included in the basket *b*_*i*_, and *σ*(*x*) is the logistic activation function to map onto probability space. To implement this objective, we sampled a number of negative items that were absent in the current basket *b*_*i*_ (in our experiment, the number of negative items was equal to the number of positive items in *b*_*i*_), and maximized the expectation probability over all products and baskets (Eq 13):

$$\begin{array}{c} \ell_{RNN}=E_{b_{i}}\left[E_{v,v^{\prime }}\left[p\left(b_{i},v≻v^{\prime }\right)\right]\right]. \end{array}$$ (13)

To process sequential data, for example for market basket recommendation, it is common to use recurrent prediction mechanisms (e.g. LSTM, GRU or vanilla RNN). While other alternatives exist, they either tend to underperform in recommendation tasks (e.g. Causal 1d convolutions), or require a large amount of good-quality data (e.g. self-attention and transformer mechanisms). Since our data does not require the prediction of extremely long sequences, using a GRU cell is a suitable design choice which balances out predictive performance with training speed.

### 3.6 Parameters optimization and cross-validation

Parameters tuning is a decisive step when building a machine learning model since most of the models are heavily reliant on their selected parameters and provide substantially better performance when properly optimized. Ignoring the optimization of parameters can lead to the selection of a sub-optimal solution at the end of the experimentation.

There exist several methods to optimize the model’s parameters such as Grid Search, Random Search or Bayesian optimization [70, 71].

Grid search takes a grid of parameters and carries out exhaustive testing with all parameter combinations in order to ultimately select the one that yields the best results for the data at hand. In this study, we used Random Search as the parameter optimization technique for the models listed in the subsection 3.5. Similarly to Grid Search, Random Search considers a grid of parameters and values. However, Random Search conducts trials on random combinations of parameters instead of performing exhaustive search. This allows one to use distributions instead of specific values for continuous settings and ensures a better time and resource management (the running time is not necessarily related to the amount of parameters/values as the number of parameter combinations to be tested can be fixed by the user). Random Search has been shown to outperform Grid Search in terms of both the results and the running time [70].

While using Random Search, we carried out cross-validation (k-fold) to ensure that the best selected model does not overfit the data [72, 73]. K-fold cross-validation is a common model validation technique in machine learning which consists in dividing the data into *k* equally sized sub-samples. A single sub-sample is then retained for validation purposes as the model is trained on the rest of the data (i.e. on the remaining *k* − 1 sub-samples). This process is repeated *k* times using each sub-sample exactly once for validation. The final evaluation of the model is the average of the *k* results. In our experiments, we set *k* = 5 (which is a commonly used number of sub-samples) [74]. Using this methodology we were able to optimize the parameters of the 10 machine learning described in the subsection 3.5, making sure that the results presented in Tables 1–3, are not due to data overfitting and correspond to real-case scenarios.

**Table 1:** F-scores provided by supervised ML/DL methods for all users of the MyGroceryTour website as well as for each of the four identified clusters of users. The best overall results are highlighted in bold.

ML/DL method	All Users	Cluster 1	Cluster 2	Cluster 3	Cluster 4
	831 users	278 users	276 users	214 users	63 users
Decision Tree	0.418	0.473	0.306	0.487	0.425
Random Forest	0.516	**0.583**	0.355	0.643	0.508
Gradient Boosting Tree	0.435	0.496	0.313	0.509	0.444
CatBoost	0.465	0.534	0.288	0.619	0.414
XGBoost	0.438	0.501	0.312	0.521	0.431
Naive Bayes	0.268	0.352	0.203	0.221	0.347
SVM-RBF	0.514	0.579	0.337	**0.662**	0.482
Logistic Regression	0.503	0.562	0.363	0.616	0.470
MLP	0.437	0.489	0.296	0.547	0.454
RNN (GRU)	**0.559**	0.568	**0.506**	0.605	**0.597**
Average F-score	0.455	0.514	0.328	0.543	0.457

**Table 2:** Recall / Sensitivity provided by supervised ML/DL methods for all users of the MyGroceryTour website as well as for each of the four identified clusters of users. The best overall results are highlighted in bold.

ML/DL method	All Users	Cluster 1	Cluster 2	Cluster 3	Cluster 4
	831 users	278 users	276 users	214 users	63 users
Decision Tree	0.602	0.625	0.481	0.714	0.596
Random Forest	0.689	**0.738**	0.497	**0.872**	0.665
Gradient Boosting Tree	0.627	0.653	0.503	0.749	0.609
CatBoost	0.605	0.661	0.390	0.824	0.530
XGBoost	0.633	0.671	0.503	0.755	0.598
Naive Bayes	0.564	0.587	0.529	0.556	0.611
SVM-RBF	0.588	0.661	0.383	0.758	0.557
Logistic Regression	0.643	0.701	0.490	0.778	0.576
MLP	0.587	0.648	0.413	0.719	0.599
RNN (GRU)	**0.729**	0.723	**0.648**	0.834	**0.754**
Average Recall	0.626	0.666	0.483	0.755	0.609

**Table 3:** Accuracy (in %) provided by supervised ML/DL methods for all users of the MyGroceryTour website as well as for each of the four identified clusters of users. The best overall results are highlighted in bold.

ML/DL method	All Users	Cluster 1	Cluster 2	Cluster 3	Cluster 4
	831 users	278 users	276 users	214 users	63 users
Decision Tree	40.5	46.5	30.1	44.8	42.4
Random Forest	49.3	**55.6**	33.3	**60.9**	48.9
Gradient Boosting Tree	41.5	47.5	30.1	47.4	42.9
CatBoost	47.4	54.8	29.8	60.3	45.3
XGBoost	41.8	48.4	29.5	48.0	42.4
Naive Bayes	34.7	42.0	26.2	35.9	40.7
SVM-RBF	50.3	56.6	33.6	63.8	46.8
Logistic Regression	47.3	53.1	33.9	57.2	44.4
MLP	42.1	47.3	29.5	50.0	44.9
RNN (GRU)	**53.3**	54.1	**48.4**	57.1	**57.8**
Average Accuracy	44.8	50.4	32.4	52.5	45.6

### 3.7 MyGroceryTour recommender system

In order to determine the products to be recommended to a given user by the MyGroceryTour recommender system, we classify all the products based on the user’s purchase history, current specials information and products availability in each of local grocery stores considered. In order to be able to classify the products efficiently, the use both positive and negative feedbacks is necessary. One personalized machine learning model (i.e. one model per user) is built and weekly updated in our system.

While we consider the products bought by a given user as positive feedback, we regard as negative feedback all the products that were available to this user at the time of the order but not acquired by him/her. For an order of size *S*, if *T* is the total number of products available to the user at the time of the order, then the negative feedback *N* for that order is *N* = *T* − *S*.

Typically, *N* represents thousands of products, while *S* usually varies from 5 to 50. This difference in size between positive and negative feedback leads to unbalanced training data and may result in a significant loss in performance. Similarly to Xia *et al* [33], we decided to use an undersampling method to balance the user’s data instead of considering negative feedback all available and disregarded items. Undersampling methods have proven to be efficient for both binary and multi-class classifications [75, 76].

As the number of products recommended by the machine learning models is often greater than an average grocery list size, *S*_*u*_, calculated for a given user *u*, for the final recommendation only the *S*_*u*_ items with the highest confidence scores were retained. Typically, the confidence score was calculated as the probability estimate for the predicted class for a given observation; it can for instance be obtained using the *predict_proba* function in scikit-learn.

## 4 Results and discussion

### 4.1 Clustering analysis

As mentioned above, we first used clustering to identify the profiles of the users of MyGroceryTour. To do so, we considered the following features:

- *avg_price* (numerical): the average price of products bought by a specific user;
- *avg_special* (numerical): the average discount percentage on products bought by a specific user;
- *avg_list_size* (numerical): the average size of the shopping list of a specific user;
- *pca_category* (numerical): this feature accounts for the category of products selected by a specific user. Here, we built a 831 × 24 matrix (831 is the number of users and 24 is the number of available categories) reflecting the user’s choice of different categories of products. Each value of this matrix represents the number of items of a given category acquired by a specific user. We carried out the PCA analysis to reduce the matrix dimension and to determine the percentage of variance accounted by the main principal axes. The first (principal) PCA axis accounted for 72.6% of the total variance, the second axis for 12.1%, whereas the variance explained by the remaining axes was negligible. We decided to keep for our clustering analysis a single transformed feature representing the product’s category. The new transformed feature corresponds to the normalized values of the first principal axis. This allows us to give the same weight to all features considered by clustering algorithms.
- *avg_fidelity_ratio* (numerical): the average of the quantity-based fidelity ratio (QFR) and the price-based fidelity ratio (PFR) defined in Eqs (14) and (15), respectively. Here, *avg*_*fr*_*u*_ = (*QFR*_*u*_ + *PFR*_*u*_)/2, where *u* is a given user, *avg*_*fr*_*u*_ is the average fidelity ratio, *QFR* is the quantity-based fidelity ratio and *PFR* is the price-based fidelity ratio.

The quantity-based fidelity ratio (QFR) and the price-based fidelity ratio (PFR) defined below are both meant to give insight on the customer’s fidelity to his/her favorite store.

The QFR value close to 1 indicates that a given consumer tends to do his/her grocery shopping in the same (favorite) store, whereas the QFR value close to 0 indicates that the customer tends to do his/her grocery shopping in many different stores. The quantity-based fidelity ratio is defined as follows:

$$\begin{array}{c} QFR_{u}=\{\begin{array}{cc} \frac{X_{max,u}}{X_{total,u}}=1, & \text{if}\;n=1\\ \frac{X_{max,u}-\frac{1}{\left(n-1\right)}\sum_{i=2}^{n}X_{i,u}}{X_{total,u}}, & \text{if}\;n>1 \end{array} \end{array}$$ (14)

where *u* represents a given user, *n* is the total number of stores where the user *u* ($n\in N^{*}$) bought at least one product, *X*_*max*,*u*_ is the total number of products acquired by the user *u* in his/her favorite store (i.e. where he/she made most of his/her purchases), and *X*_*total*,*u*_ ($X_{total,u}=X_{max,u}+\sum_{i=2}^{n}X_{i,u}$) is the total number of products purchased by the user *u* over all the stores where he/she bought at least one product.

Similarly, the price-based fidelity ratio (PFR) depends on a total price of the products acquired by the customer in his/her favorite store. The price-based fidelity ratio is defined as follows:

$$\begin{array}{c} PFR_{u}=\{\begin{array}{cc} \frac{P_{max,u}}{P_{total,u}}, & \text{if}\;n=1\\ \frac{P_{max,u}-\frac{1}{\left(n-1\right)}\sum_{i=2}^{n}P_{i,u}}{P_{total,u}}, & \text{if}\;n>1 \end{array} \end{array}$$ (15)

where *u* represents a given user, *n* is the total number of stores where the user *u* ($n\in N^{*}$) bought at least one product, *P*_*max*,*u*_ is the total price of all products acquired by the user *u* in his/her favorite store, and *P*_*total*,*u*_ ($P_{total,u}=P_{max,u}+\sum_{i=2}^{n}P_{i,u}$) is the total price of all products purchased by the user *u* over all the stores where he/she bought at least one product.

The input data for clustering analysis consisted of a matrix of 831 observations (i.e. corresponding to the 831 selected users of MyGroceryTour) and 5 features. Prior to performing clustering, we normalized the data at hand. We tested both Z-score and MinMax normalizations. The results presented below have been obtained using MinMax normalization as it provided slightly better clustering results than Z-score. The clustering analysis was carried out using both the Ward algorithm [51], which is one of the most popular hierarchical clustering algorithms, and K-means [50], which is certainly the most popular partitioning algorithm, through their scikit-learn implementations. The default scikit-learn parameters of the Ward and K-means algorithms were used.

We used the popular Silhouette [77] and Davies-Bouldin (DB) [78] cluster validity indices to determine the number of clusters in our dataset.

The Silhouette width is defined as follows. Given a partition *P* of a data set *X* with *N* objects, the Silhouette width *s*(*x*_*i*_), for object *x*_*i*_ ∈ *X*, represents the degree of correspondence between *x*_*i*_ and the partition. The average distance from object *x*_*i*_ to its cluster *C*_*k*_ can be defined as follows (Eq 16):

$$\begin{array}{c} a\left(i\right)=\frac{1}{|C_{k}|}\sum_{j\in C_{k}}\sqrt{d(x_{i},x_{j})}, \end{array}$$ (16)

and the distance to a nearest object in another cluster as follows (Eq 17):

$$\begin{array}{c} b\left(i\right)=\underset{C_{k}:x_{i}\notin C_{k}}{\text{min}}\{\frac{1}{|C_{k}|}\sum_{j\in P_{k}}\sqrt{d(x_{i},x_{j})}\}. \end{array}$$ (17)

The Silhouette width for an object *s*(*x*_*i*_) is defined as the relative difference between *a*(*x*_*i*_) and *b*(*x*_*i*_) (Eq 18):

$$\begin{array}{c} s\left(x_{i}\right)=\frac{b\left(x_{i}\right)-a\left(x_{i}\right)}{\text{max}\{a\left(x_{i}\right),b\left(x_{i}\right)\}}. \end{array}$$ (18)

The global Silhouette width value is then defined as follows (Eq 19):

$$\begin{array}{c} s\left(P\right)=\frac{1}{N}\sum_{x_{i}\in X}\left(x_{i}\right). \end{array}$$ (19)

It represents the extent of consistency of partition P. The maximum value of *s*(*P*) corresponds to the “right” number of clusters.

The Davies-Bouldin index is the average similarity between each cluster *C*_*i*_ for *i* = 1, …, *k* and its most similar counterpart *C*_*j*_. It is calculated as follows (Eq 20):

$$\begin{array}{c} DB=\frac{1}{k}\sum_{i=1}^{k}\underset{i\neq j}{max}\;S_{ij}, \end{array}$$ (20)

where *S*_*ij*_ is the similarity value between clusters, calculated as (*d*_*i*_ + *d*_*j*_)/*δ*_*ij*_, where *d*_*i*_ are *d*_*j*_ are the the mean distances between the objects in cluster *C*_*i*_ and *C*_*j*_, respectively, and the cluster centroids, and *δ*_*ij*_ is the distance between the centroids of clusters *C*_*i*_ and *C*_*j*_. The minimum value of the DB index corresponds to the “right” number of clusters.

**Fig 7:**
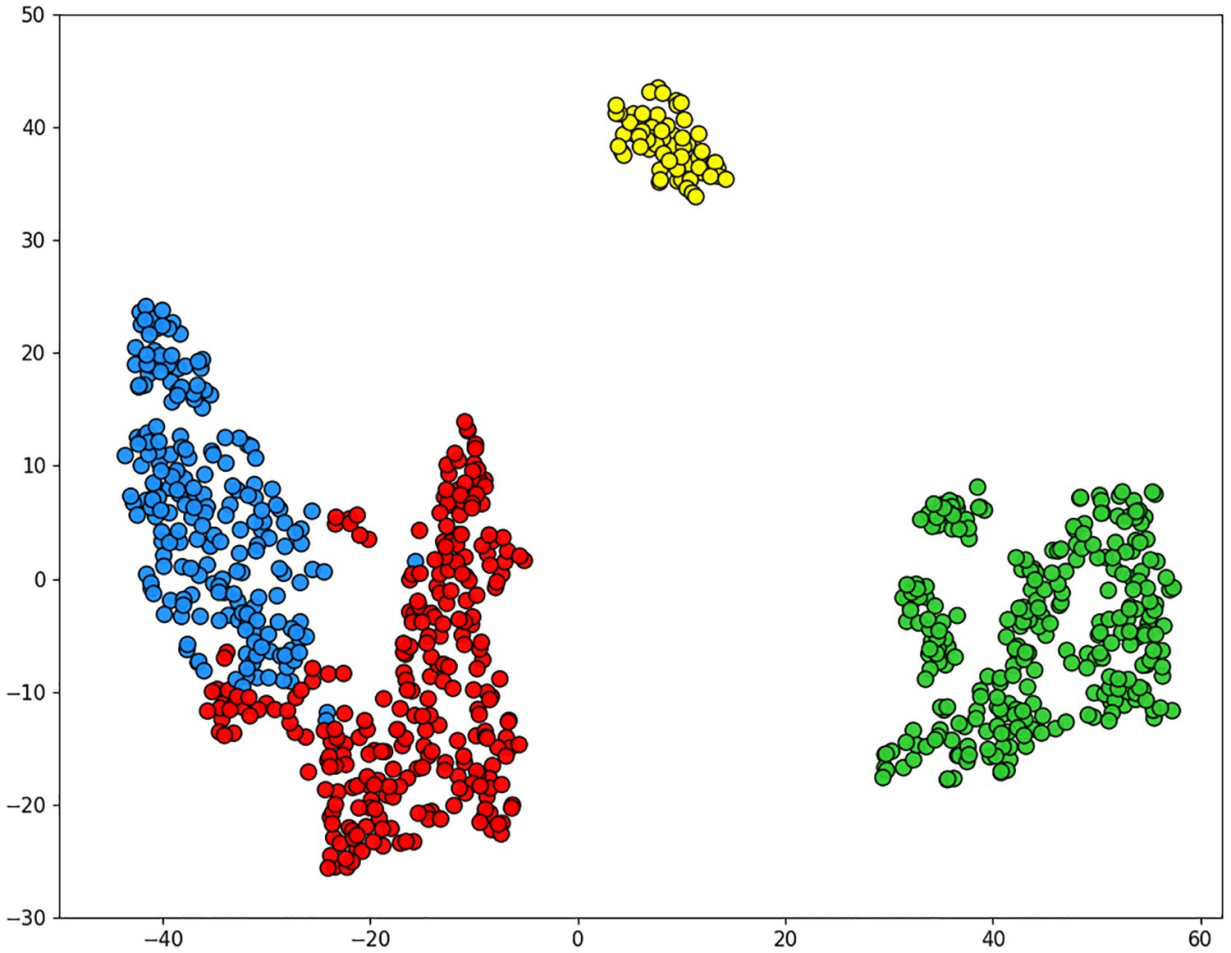
Clustering results: Clustering solution provided by the Ward algorithm for K = 4 clusters (i.e best number of clusters according to the Silhouette and Davies-Bouldin cluster validity indices). Dimentionality reduction after clustering was performed by t-SNE (for visualization purposes). The 4 clusters of customers found by hierarchical Ward-based clustering are represented by different colors (Cluster 1 of 278 users—in red, Cluster 2 of 276 users—in green, Cluster 3 of 214 users—in blue, Cluster 4 of 63 users- in yellow.

**Fig 8:**
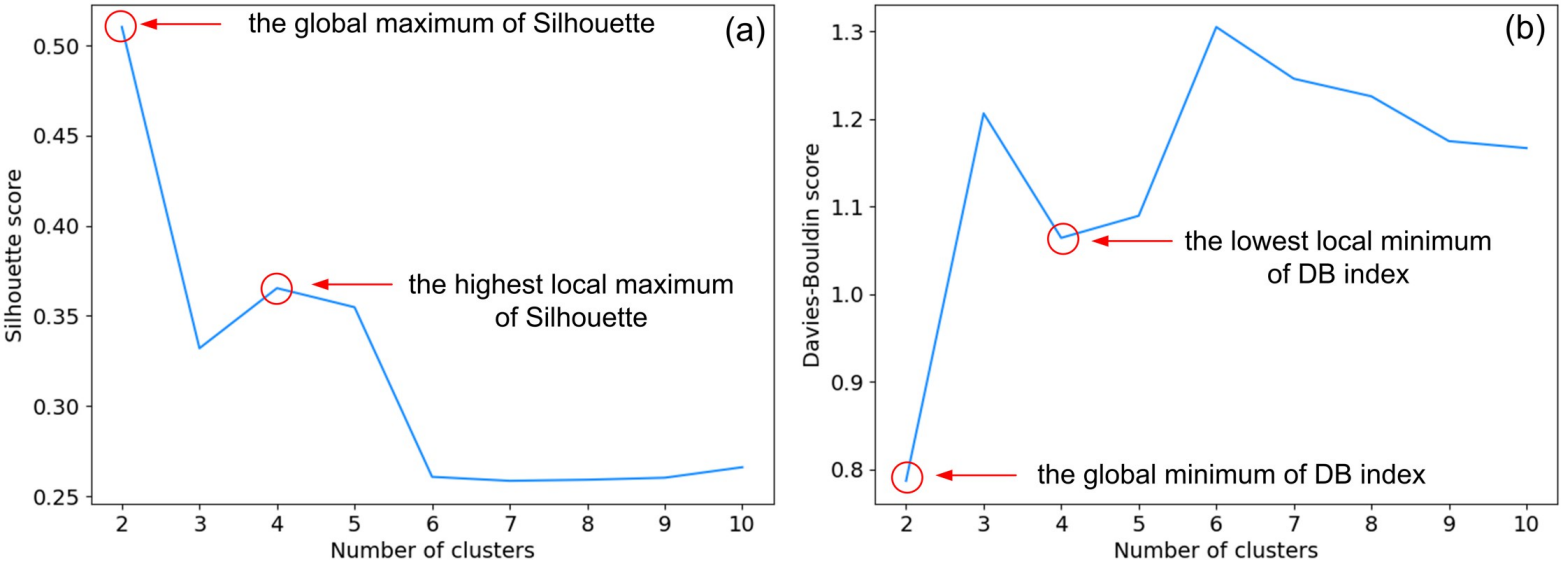
Silhouette and Davies-Bouldin cluster validity scores variation with respect to the number of clusters.

While the highest value of the Silhouette and the lowest value of the Davies-Bouldin indices were found for the solution with *K* = 2 clusters, we present here the most interesting solution found for *K* = 4 clusters (see Fig 7). This solution corresponds to the highest local maximum of the Silhouette and the lowest local minimum of the Davies-Bouldin indices (see Fig 8). We used the t-distributed Stochastic Neighbor Embedding (tSNE) [79] as a dimensionality reduction method to visualize the clustering solution provided by the Ward algorithm (see Fig 7). During our experiments, we used the perplexity of 30 and the learning rate of 925 as parameters for the tSNE method, whereas the tSNE initialization parameter was based on principal component analysis [80] in order to preserve the general shape of the data.

It is worth mentioning that the clustering solution provided by K-means (for *K* = 4 clusters; this solution is not presented here) was similar, but had a slightly more important cluster overlap, compared to that found by Ward. The four user profiles shown in Fig 7 are as follows:

- Cluster 1 (in red in Fig 7) includes customers who are moderately sensible to specials and usually buy their groceries in the same store (i.e, have high fidelity ratios);
- Cluster 2 (in green in Fig 7) is the most diverse cluster that consists of customers buying their groceries in different stores (i.e., have low fidelity ratios). The members of this cluster are usually, sensitive to specials;
- Cluster 3 (in blue in Fig 7) comprises customers who usually purchase the same (or similar) products in the same store (i.e. have high fidelity ratios), almost not reacting to specials;
- Cluster 4 (in yellow in Fig 7) includes customers who are very sensitive to specials and buy their groceries in the same store (i.e., have high fidelity ratios).

### 4.2 Application and comparison of supervised machine learning algorithms

To assess the performance of the 10 traditional machine learning and deep learning algorithms considered in our study, we used F-score, which is a popular and reliable metric used to evaluate classification methods [81–83]. F-score is the harmonic mean of the precision and recall. It is defined as follows (Eq 21):

$$\begin{array}{c} F=\frac{2\times Precision\times Recall}{Precision+Recall}, \end{array}$$ (21)

where the recall is defined as $\frac{TP}{TP+FN}$ and the precision as $\frac{TP}{TP+FP}$, and TP are true positives (correctly classified positive samples), TN are true negatives (correctly classified negative samples), FP are false positives (negative sample classified as positives), and FN are false negative (positive samples classified as negatives).

### 4.3 Results

The F-score results for the traditional machine learning and deep learning algorithms considered in our study are presented in Table 1. In this table, the overall average F-score results (obtained over all 831 users of MyGroceryTour) are presented along with cluster performances.

We can observe that three algorithms stand out by outperforming the rest of the methods, providing the best F-score performance for at least one cluster of users. The best overall result consisting in F-score of 0.559 was yielded by our RNN-GRU model. This model also provided the best average results for the users from Cluster 2 (with F-score of 0.506) and those of Cluster 4 (with F-score of 0.597), whose behavior is the most difficult to predict. Random Forest returned the best results for the users of Cluster 1 (with F-score of 0.583), whereas the radial basis SVM provided the best results for the users of Cluster 3 (with F-score of 0.662; the behaviour of the users from this cluster was the easiest to predict). We can also notice that baseline algorithms such as Naive Bayes and Decision Tree consistently underperformed across all clusters.

These promising performance of the generalized RNN-GRU DREAM model, which learns a dynamic representation of a given user and captures global sequential characteristics existing among the user’s baskets, best suited to personalized basket recommendation task. It is capable of modeling the behaviour of the most diverse group of users, i.e., those forming Cluster 2, who buy their groceries in different stores and are sensitive to specials.

Tables 2 and 3 present respectively the Recall and Accuracy results provided by the ML and DL algorithms considered in our study. These results are usually concordant with the F-score results reported in Table 1 as in both cases the RNN-GRU algorithm outperforms the other methods for the whole set of 831 users.

**Fig 9:**
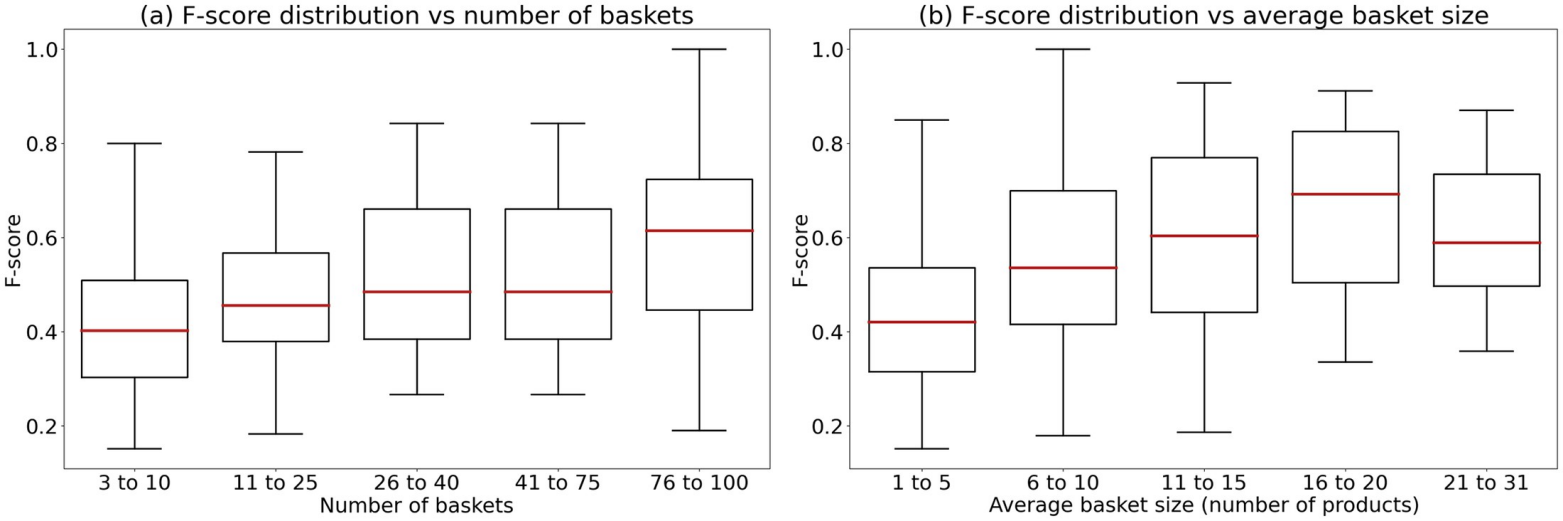
Boxplots constructed for the Random Forest prediction results: (a) F-score variation with respect to the number of baskets; (b) F-score variation with respect to the number of products per baskets.

**Fig 10:**
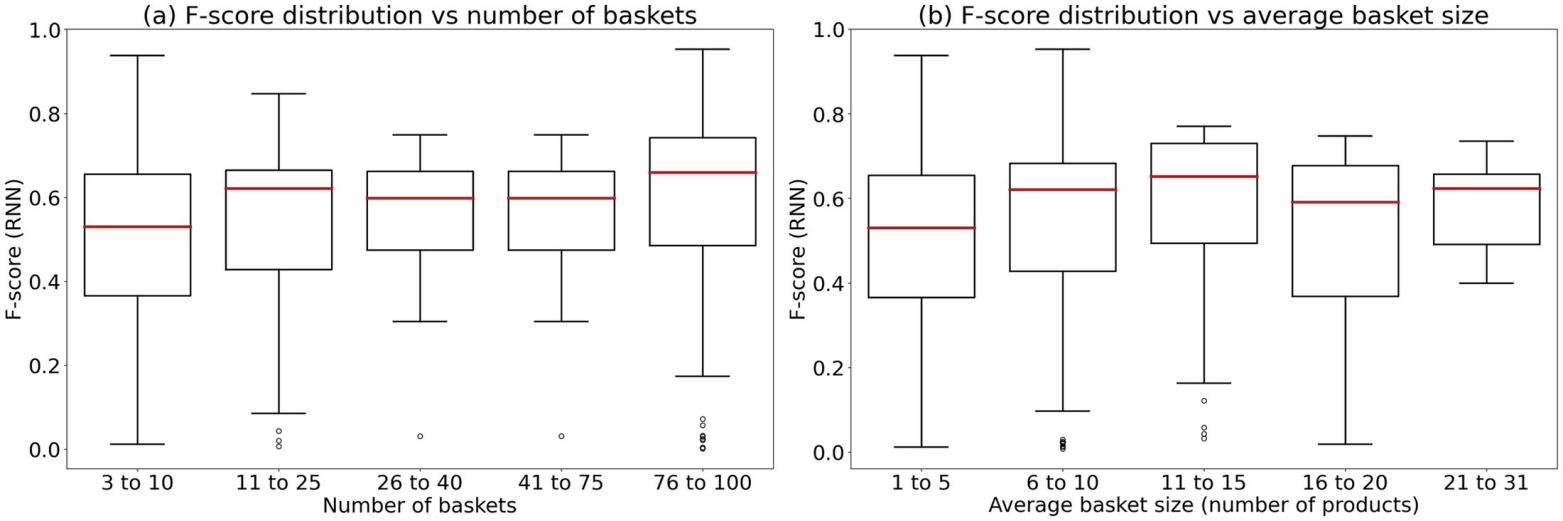
Boxplots constructed for the RNN (GRU) prediction results: (a) F-score variation with respect to the number of baskets; (b) F-score variation with respect to the number of products per baskets.

Figs 9 and 10 illustrate the impact of the number of baskets and the average basket size on the prediction performance of Random Forest (the best traditional machine learning algorithm) and RNN-GRU (the best deep learning algorithm), respectively. We can observe that both Random Forest and RNN-GRU work best for users with high numbers of baskets (75 and greater), although the impact of the number of baskets is more important for Random forest (see Fig 9a).

On the other hand, a larger average basket size does not always results in a better prediction performance. For example, Random Forest (see Fig 9b) is less effective for users with an average basket size over 20 items than for those with an average basket size varying from 16 and 20 items. This could be due to complex relationships between items within the baskets. The performance of RNN-GRU seems to be less affected by the basket size, although this algorithm works better for users having more than 5 items in their baskets on average.

## 5 Conclusion

**Fig 11:**
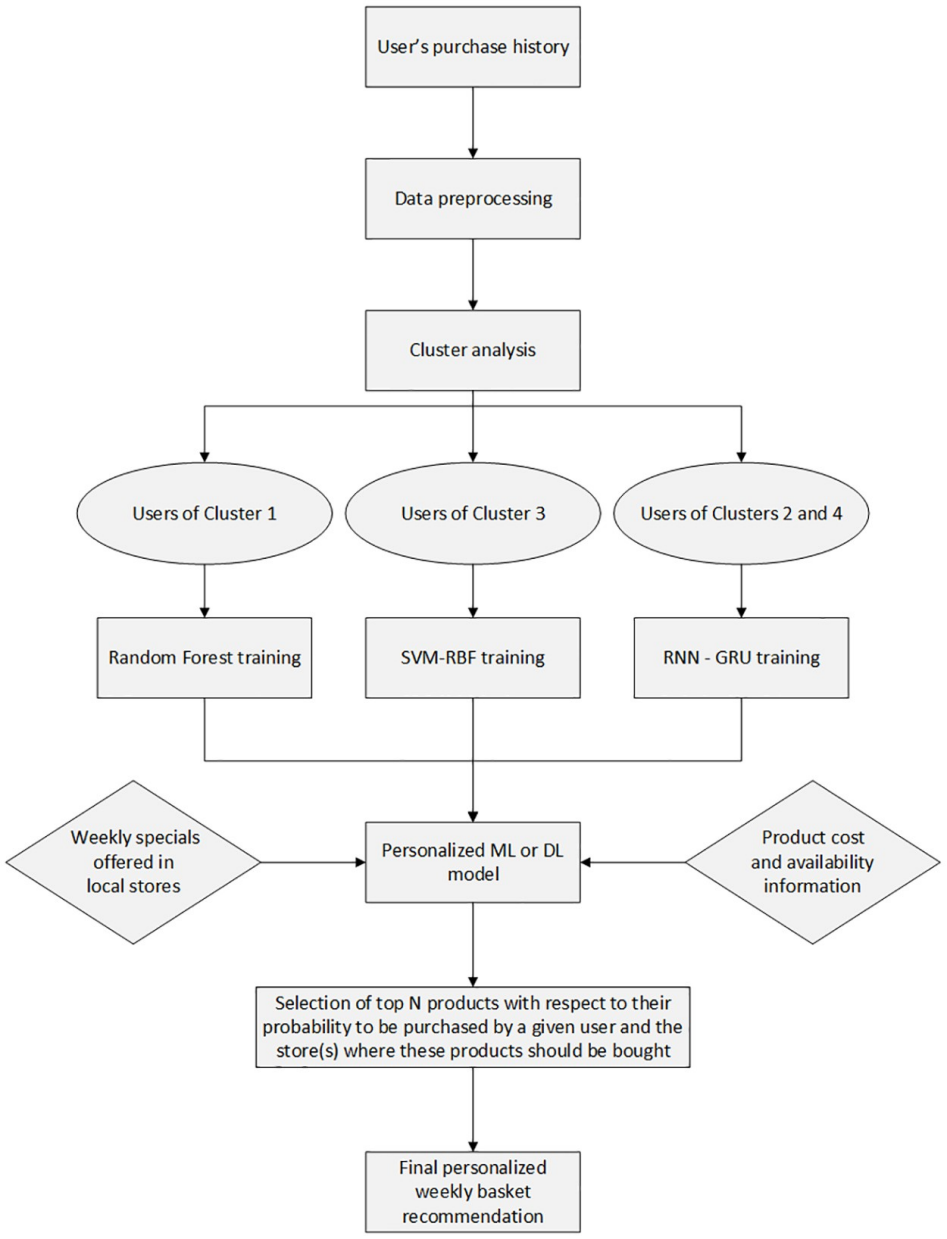
Overview of the proposed recommendation framework.

In this paper, we presented a novel personalized Recommender System included in the MyGroceryTour web platform, which is designed to suggest the best weekly grocery deals to Canadian customers. Our system applies the most appropriate ML or DL prediction model (see Fig 11) to provide a given customer with a weekly grocery list that suits him/her best as well as the list of stores in which the customer should purchase each product being recommended. Our system takes into account several features related to the customer’s purchase history as well as features related to the current price and availability of products in local grocery stores. One of the advantages of our Recommender System is that it can recommend to each customer the products he/she has never bought before, which can be helpful to discover new relevant products or be aware of limited-time deals.

Our results demonstrate that different ML and DL methods should be applied for different clusters of users (see the results in Tables 1–3). To identify these groups of users according to their shopping behavior, we carried out two representative clustering algorithms, K-means (partitioning algorithm) and Ward (hierarchical clustering algorithm), which are known for their simplicity and speed. Our clustering analysis, conducted using Ward’s algorithm, divided the entire group of 831 Canadian customers considered in this work into four clusters according to their shopping habits. In this study, we also introduced the average fidelity ratio feature used in our clustering analysis. This feature was defined as the average of the quantity-based fidelity ratio and the price-based fidelity ratio (PFR) introduced via Eqs (14) and (15), respectively. We then used different traditional machine learning and a new deep learning models to provide next basket recommendations. Interestingly, the average F-score values obtained for users from different clusters were quite different (see Table 1). They varied from 0.328 (for users of Cluster 2—who buy their groceries in different stores, and are sensitive to specials) to 0.543 (for users of Cluster 3—who usually purchase the same, or similar, products in the same store, and are not very sensitive to specials). We can also observe that some of ML methods were much better than others in recommending items for a specific cluster of users. Thus, it would be plausible to apply different prediction methods for different groups of customers: Random Forest for customers from Cluster 1, RNN-GRU for customers from Clusters 2 and 4, and SVM-RBF for customers from Cluster 3. Overall, the best results were provided by our RNN-GRU implementation. In terms of the average F-score, it outperformed Random Forest, the second best performing model, by 0.043. RNN-GRU also yielded the most consistent results across all clusters. The flowchart presented in Fig 11 provides a general overview of our Recommender System.

The superiority of the proposed RNN-GRU model indicates that in a grocery shopping context, temporal behaviour of the user, which reveals the user’s dynamic interests at different times, and sequential characteristics of shopping baskets, which reflect interactions between all user’s baskets over time, are two crucial prediction factors for next basket recommendation. Our promising prediction results can be explained by the nature of the data: indeed, grocery data are often very repetitive as users tend to buy a core of similar items (such as first necessity products) regularly, thus developing constant habits.

It is important to note that in terms of F-score our personalized RNN-GRU model outperformed the recent general LSTM-based model proposed by Tahiri *et al*. [6] by 0.339 when we used the new data available on the MyGroceryTour platform. Furthermore, for the augmented data considered by Tahiri *et al*., our F-score result was 0.189 higher than that of Tahiri and co-authors. The model introduced in our study is personalized (i.e. the model’s parameters are tuned for each user). Specifically, our current model is equivalent to training a single aggregate model (as that of Tahiri *et al*.) for all users, and conditioning the inputs on the user embedding. Thus, in our current model, the implicit user embedding is the ground-truth one-hot vector. This explains its superior performance compared to the aggregate model of Tahiri *et al*. The LSTM models tend to be heavier for inference and training needs than GRUs, which is a limiting factor in our use-case. However, it is indeed possible to swap out one for the other in most practical setting, when the sequence length is not too large.

Table 4 reports the prediction performances of the most important recent ML and DL models used in the field of next basket recommendation. We can see that it’s difficult to compare directly our results to those provided by most of the existing studies (as well as to compare the results of the existing studies among them) because most of these studies have been conducted using different datasets and different evaluation metrics. The only direct comparison can be done with the work of Tahiri et al. (2019), as these authors also analyzed MyGroceryTour data (see above). One of the main contributions of our study, in the addition to the use of clustering, is that we work in the multi-class (i.e. multi-store) classification context, while all previous studies considered the case of binary (i.e. one-store) classification, i.e. when a product can be recommended or not without suggesting the store where it should be bought (if recommended).

**Table 4:** Comparison of the performance of some recent ML and DL models used for next basket recommendation.

Study	Year	Classification type	Model	Accuracy	F-score	Recall	nDCG	Data
Yu et al. (Ta-Feng)	2016	Binary	DREAM	N/A	0.070	N/A	0.086	Two real-world
Yu et al. (Ta-Feng)	2016	Binary	TOP	N/A	0.045	N/A	0.072	datasets
Yu et al. (Ta-Feng)	2016	Binary	NMF	N/A	0.054	N/A	0.075	(Ta-Feng^1^, T-mall^2^)
Yu et al. (Ta-Feng)	2016	Binary	MC	N/A	0.052	N/A	0.075	
Yu et al. (Ta-Feng)	2016	Binary	FPMC	N/A	0.059	N/A	0.082	
Yu et al. (Ta-Feng)	2016	Binary	HRM	N/A	0.069	N/A	0.084	
Yu et al. (T-mall)	2016	Binary	DREAM	N/A	0.073	N/A	0.173	
Yu et al. (T-mall)	2016	Binary	TOP	N/A	0.018	N/A	0.040	
Yu et al. (T-mall)	2016	Binary	NMF	N/A	0.043	N/A	0.110	
Yu et al. (T-mall)	2016	Binary	MC	N/A	0.024	N/A	0.048	
Yu et al. (T-mall)	2016	Binary	FPMC	N/A	0.057	N/A	0.130	
Yu et al. (T-mall)	2016	Binary	HRM	N/A	0.069	N/A	0.159	
Xia et al.	2017	Binary	RF	N/A	0.850	0.780	0.969	Anonymized coupon
Xia et al.	2017	Binary	XGBoost	N/A	0.800	0.830	0.857	data
Che et al. (Ta-Feng)	2019	Binary	IIAAN	N/A	0.134	0.159	0.158	Three real-world
Che et al. (Taobao)	2019	Binary	IIAAN	N/A	0.022	0.034	0.021	datasets (Ta-Feng^1^,
Che et al. (JingDong)	2019	Binary	IIAAN	N/A	0.164	0.295	0.157	Taobao^3^, JingDong^4^)
Tahiri et al. (MyGroceryTour data)	2019	Binary	LSTM, NNMF, GBT	27%	0.220	0.510	N/A	MyGroceryTour data (for Canada, 2019)
Tahiri et al. (augmented MyGroceryTour data)	2019	Binary	LSTM, NNMF, GBT	49%	0.370	0.700	N/A	Augmented MyGroceryTour data (2019)
Le et al. (Ta-Feng)	2019	Binary	LSTM	N/A	0.064	N/A	N/A	Three real-world
Le et al. (Delicious)	2019	Binary	LSTM	N/A	0.050	N/A	N/A	datasets (Ta-Feng^1^,
Le et al (Foursquare).	2019	Binary	LSTM	N/A	0.036	N/A	N/A	Delicious^5^, Foursquare^6^)
Lee et al. (Instacart)	2020	Binary	LSTM-CUMMP	N/A	0.194	N/A	N/A	Instacart^7^ (2017)
Faggioli et al. (Dunnhumby)	2020	Binary	UP-CF	N/A	N/A	N/A	0.212	Two real-world grocery datasets:
Faggioli et al. (Instacart)	2020	Binary	UP-CF	N/A	N/A	N/A	0.429	Dunnhumby^8^ and Instacart^7^ (2017)
Zheng and Ding (Book-Crossing)	2022	Binary	IGNN	N/A	N/A	0.275	N/A	Three real-world datasets:
Zheng and Ding (Yelp)	2022	Binary	IGNN	N/A	N/A	0.149	N/A	Book-Crossing^9^,
Zheng and Ding (Foursquare)	2022	Binary	IGNN	N/A	N/A	0.204	N/A	Yelp^10^, Foursquare^6^
Our study (MyGroceryTour data)	2022	Multi-class (multi-store)	RNN-GRU (extended DREAM)	53.3%	0.559	0.729	N/A	MyGroceryTour data^11^ (for Canada, 2022)

^1^ Ta-Feng dataset url: https://www.kaggle.com/datasets/chiranjivdas09/ta-feng-grocery-dataset

^2^ Tmall dataset url: https://tianchi.aliyun.com/dataset/dataDetail?dataId=53

^3^ Taobao dataset url: https://www.kaggle.com/datasets/pavansanagapati/ad-displayclick-data-on-taobaocom

^4^ JingDong dataset url: https://www.datafountain.cn/competitions/247/datasets

^5^ Delicious dataset url: https://grouplens.org/datasets/hetrec-2011/

^6^ Foursquare dataset url: https://sites.google.com/site/yangdingqi/home/foursquare-dataset

^7^ Instacart dataset url: https://www.kaggle.com/c/instacart-market-basket-analysis

^8^ Dunnhumby dataset url: https://www.kaggle.com/datasets/frtgnn/dunnhumby-the-complete-journey

^9^ Book-Crossing dataset url: http://www2.informatik.uni-freiburg.de/~cziegler/BX/

^10^ Yelp dataset url: https://www.yelp.com/dataset

^11^ MyGroceryTour dataset url: https://drive.google.com/file/d/1q-LkWMx5ar-OGlPPLFwSDi-IbLe7ZaIo/view?usp=sharing

The Python implementation of all clustering and machine learning algorithms used in our work as well as the described anonymized 831-user data set are available in our GitHub repository at: https://github.com/Achrafb11/Smartshopping.

One of the limitations of our approach lies within the platform itself. Indeed, MyGroceryTour does not allow people to buy the products directly. Thus, we have no assurance that the users actually bought the items included in their grocery lists. We also cannot track stocks in different stores to potentially notify the users of shortages prior to adding products to their grocery lists. Our Recommender System is also sensitive to the cold start problem and it is not yet able to predict the exact quantity of each item recommended for inclusion to the user’s next basket. We plan on addressing these limitations in our future work, in which we will also explore the impact of seasonality on grocery shopping habits, which could lead to improved recommendations as well.
